# The rise in mortality due to intentional self-poisoning by medicines in Brazil between 2003 and 2022: relationship with regional and global crises

**DOI:** 10.3389/fpubh.2024.1428674

**Published:** 2024-07-11

**Authors:** Maximiliano Loiola Ponte Souza, Jesem Douglas Yamall Orellana, Francimar Oliveira Jesus, Bernardo Lessa Horta

**Affiliations:** ^1^Technical Office, Oswaldo Cruz Foundation, Eusébio, Brazil; ^2^Leonidas and Maria Deane Institute, Oswaldo Cruz Foundation, Manaus, Brazil; ^3^Postgraduate Program in Epidemiology, Federal University of Pelotas, Pelotas, Brazil

**Keywords:** mortality, suicide, poisoning, health inequities, Brazil

## Abstract

In recent years, suicide rates in Brazil have increased, but little is known about the temporal behavior and characteristics of suicides due to intentional self-poisoning by medicines. The aim of the present study was to provide an overview of sociodemographic characteristics and place of death related to suicide due to intentional self-poisoning by medicines, to evaluate the trend of mortality rates in Brazil between 2003 and 2022, and its relationship with regional and global crises. Ecological time series study with data from the Mortality Information System of the Brazilian Ministry of Health, related to individuals aged 10 years and over, who committed suicides due to intentional drug overdose, in the period from 2003 to 2022. The analyses were performed in the R environment in RStudio. Between 2003 and 2022, there was a predominance of deaths in women (55.5%), individuals aged 30–49 years (47.2%), of White race/color (53.2%), occurring in health facilities (67.0%), using drugs or unspecified substances (40.4%); a higher concentration in the southern region (22.8%) and a positive trend in mortality rates due to intentional drug overdose, especially from 2016 onwards. A rise of 264% was observed in the comparison of 2022 and 2003. A peculiar sociodemographic profile was observed in the victims of intentional self-poisoning by medicines and a positive temporal trend in mortality rates, especially in a period marked by regional and global crises.

## Introduction

Suicide remains a serious public health problem on a global scale, especially in developing countries (1). This is due to the fact that the implementation of preventive strategies aimed at promoting mental health and reducing suicide rates has historically been challenging, something that seems to have become even clearer during the COVID-19 pandemic (2).

In Latin America, between 2015 and 2019, about 100,000 deaths per year were due to suicide. This period also coincided with the increase in mortality rates in the region, reaching values close to 10 per 100,000 inhabitants in 2019 (1). In Brazil, a significant increase in suicide rates was also observed, especially after 2015 (3, 4).

Different methods can be used for suicide and the choice of the method can determine the lethality of the act of self-aggression. The method can be associated with cultural and sociodemographic factors as well as access to means for self-aggression (5). Despite the undeniable scientific advances regarding the control and treatment of numerous diseases in the last five decades, including mental health diseases, there has also been substantial pharmaceuticalization of daily life and suffering (6), a key concept to better understanding the substantial increase in the medicines usage related suicides. Although the main uses of medicines are for the control and treatment of diseases, they can also be used to carry out suicide. In Brazil, the largest and most populous country in South America, among all deaths due to medicines intoxication, suicide was the leading cause of mortality (7, 8).

Despite the extensive number of analyses regarding suicide rates on a global, regional and national scale, studies that explore the characteristics, rates and spatio-temporal distribution of method-specific suicide are relatively scarce (7–9), especially in relation to intentional self-poisoning by medicines, which can be prevented by restricting/controlling access to these substances (10). Therefore, increasing knowledge about specific methods that result in suicides due to intentional self-poisoning by medicines can contribute to the improvement of prevention strategies. The aim of the present study was to provide an overview of sociodemographic characteristics and place of death related to suicide due to intentional self-poisoning by medicines, to evaluate the trend of mortality rates for this method-specific suicide in Brazil between 2003 and 2022, and its relationship with regional and global crises.

## Methods

### Study type, data sources, and units of analysis

This study is an ecological time series study (Morgenstern, 1995). Data for 2003 to 2022 was obtained from the Mortality Information System of the Brazilian Ministry of Health (11). Data from 2003 to 2022 are considered revised and suitable for publication and dissemination by the Ministry of Health. The data for the resident population are estimates obtained by the Brazilian Institute of Geography and Statistics (IBGE), with retro-projection for the period from 2003 to 2010 and projection for the period from 2011 to 2022 (12). Individual mortality records were aggregated at the country level, due to there being no deaths or a reduced annual number of deaths per state or city, especially at the beginning of the time series and/or in less populated regions of the country.

### Working definitions

Since the occurrence of suicide is very low among children, we restricted the analysis to people aged 10 years or older, according to the victims’ place of residence and date of death. All records coded as intentional self-poisoning by medicines (codes X60; X61; X63; X64), according to the International Statistical Classification of Diseases and Related Health Problems (ICD-10) (13), were considered in this analysis. Finally, annual mortality rates were estimated by dividing the frequency of suicides by the estimated population, and then multiplying by 1.000.000 inhabitants.

### Included variables

The following variables were considered: gender (female; male); age group in years (10–19; 20–29; 30–49; 50–64; 65 and over); race/color (White; Indigenous/Black; others; no answer/unknown - due to the small number of victims classified in the Yellow and indigenous categories, both were aggregated in the “Others” category), marital status (single; married/common-low marriage; widowed/divorced; no answer/unknown), place of death or physical area where the death occurred (health facilities; home; public byway; others; no answer/unknown - the “Others” category is not detailed in the microdata of the Brazilian Ministry of Health); category CID-10 (X60) [intentional self-poisoning by and exposure to nonopioid analgesics, antipyretics and antirheumatics]; X61 [intentional self-poisoning by exposure to antiepileptic, sedative-hypnotic, antiparkinsonism and psychotropic drugs, not elsewhere classified]; (X63) [intentional self-poisoning by exposure to other medicines acting on the autonomic nervous system]; (X64) [intentional self-poisoning by medicines exposure to other unspecified drugs, medications and biological substances]; Region of residence (southeast; south; northeast; central-west; north), period of death (2003–20,015, 2016–2022).

### Data analysis

We analyzed the trend of observed mortality rates by visually inspecting their pattern over time. In addition, we used a locally weighted nonparametric regression (Loess) (14) to evaluate the general trend of the sequence of observations of this series over time, which can be interpreted as a smoothing of y (mortality rates) given x (year of death), with a smoothed curve and its predicted values the significance level was set to 0.05. After the decomposition of the time series and removal of the trend, the independence of the residuals of the series was evaluated using the graph of the autocorrelation function (ACF).

Additionally, ratios of the mortality rates were evaluated, taking as a reference or baseline the first observation of the time series (2003 rate) and estimated confidence intervals where the significance level was set to 0.05, in order to quantify differences.

The analyses were performed in the software R, version 4.4.0 and RStudio, version 2024.04.0 + 735 (https://www.r-project.org).

## Results

Between 2003 and 2022, 9,123 deaths were recorded due to intentional self-poisoning by medicines. These deaths were more frequent in women (55.5%), in the age group of 30–49 years (47.2%), of race/color White (53.2%); in singles (52.4%); occurring preferentially in health facilities (67.0%); and in the southeast (41.7%) and south (22.8%) of Brazil (Table 1).

**Table 1:** Sociodemographic features of suicides due to intentional self-poisoning by medicines*, 2003 to 2022, Brazil.

Covariate	*n*	%
**Sex**		
Female	5,059	55.5
Male	4,064	44.5
**Age groups (years)**		
10–19	728	8.0
20–29	1,833	20.1
30–49	4,307	47.2
50–64	1,681	18.4
65 e mais	574	6.3
**Race/Skin color**		
White	4,852	53.2
Indigenous/Black	3,928	43.1
Others**	66	0.7
No answer/Unknown	277	3.0
**Marital status**		
Single	4,779	52.4
Married/Common-law marriage	2,506	27.5
Widowed/Divorced	1,195	13.1
No answer/Unknown	643	7.0
**Place of death**		
Healthcare facilities	6,122	67.0
Household	2,317	25.4
Public byway	170	1.9
Others	490	5.4
No answer/Unknown	24	0.3
ICD-10 code***		
X60	212	2.3
X61	3,682	40.4
X63	228	2.5
X64	5,001	54.8
**Region of residence**		
Southeast	3,808	41.7
South	2,075	22.8
Northeast	1,971	21.6
Central-west	932	10.2
North	337	3.7
**Period of death**		
2003–20,015	4,381	48.0
2016–2022	4,742	52.0

*n = 9,061.

** Yellow and Indigenous people.

***X60 - Intentional overdose by exposure to nonopioid analgesics, antipyretics and antirheumatics; X61 - Intentional overdose by exposure to antiepileptic, sedative-hypnotic, antiparkinsonism and psychotropic drugs, not classified elsewhere; X63 - Intentional overdose by exposure to other drugs acting on the autonomic nervous system; X64 - Intentional overdose by exposure to other unspecified drugs, medications and biological substances.

**Figure 1:**
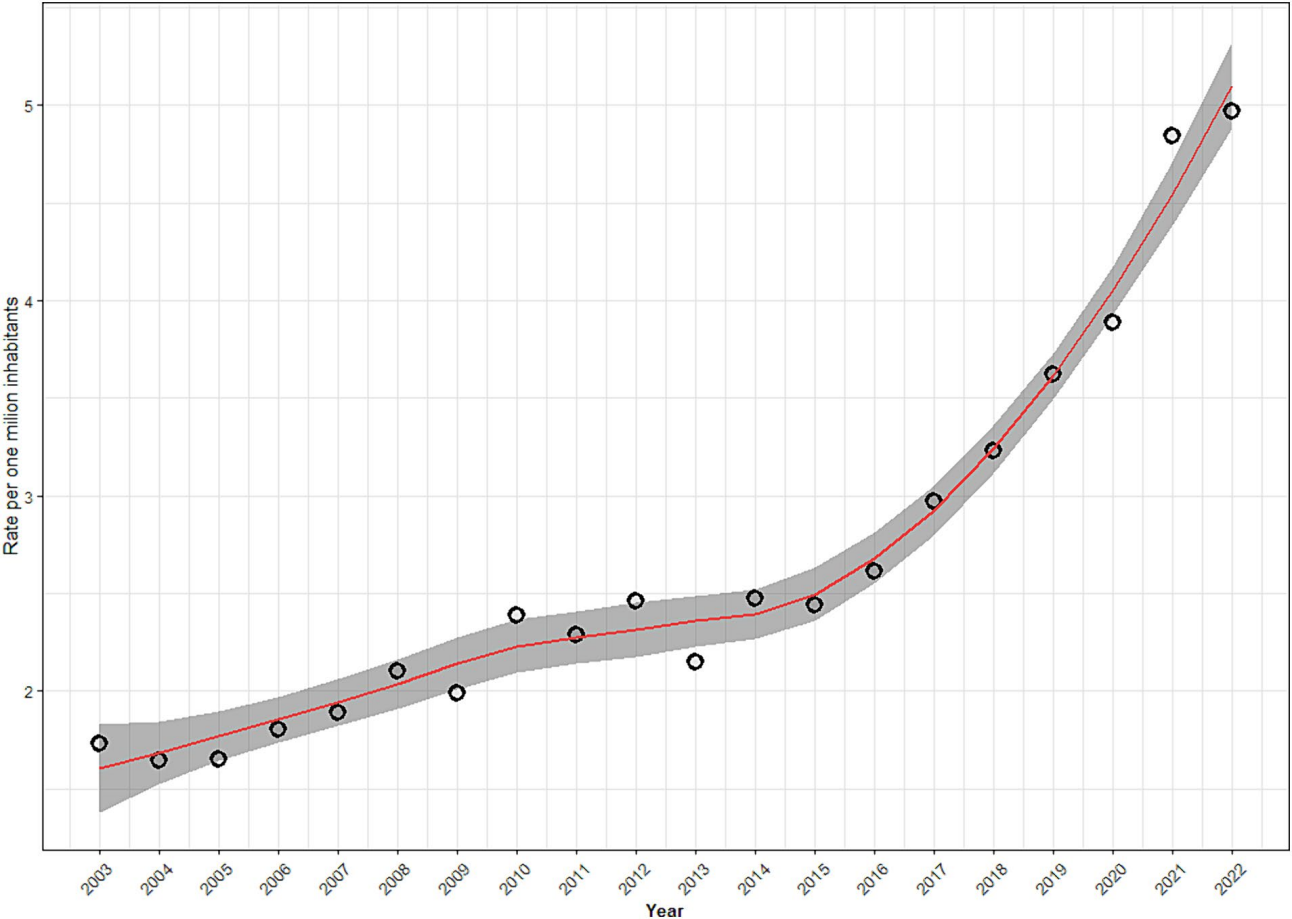
Distribution of suicide rates due to intentional self-poisoning by medicines *** and estimated curve with its respective confidence interval (95%), 2003 to 2022, Brazil.

**Figure 2:**
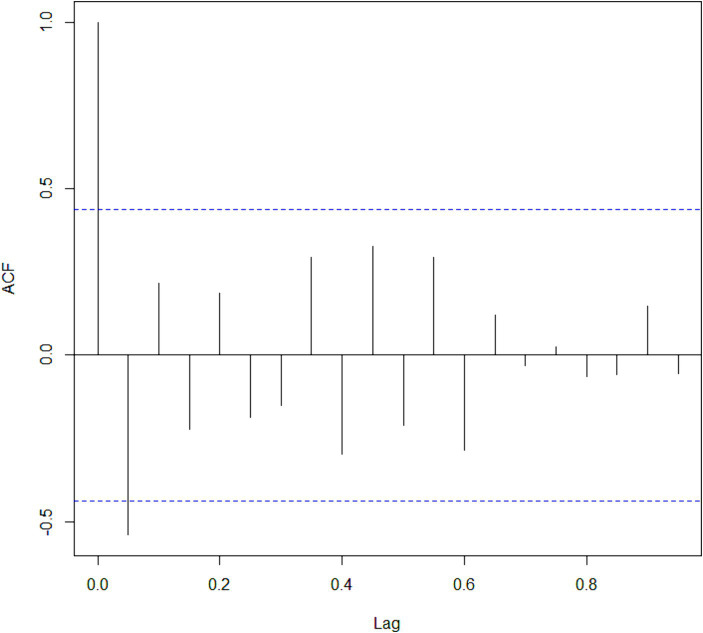
Representation of serial autocorrelation of the nonparametric regression model, 2003 to 2022, Brazil. ACF, autocorrelation function; Lag, lag range to identify noise.

According to Figure 1, mortality rates due to intentional self-poisoning by medicines increased between 2003 and 2022. In the period from 2003 to 2009, a slight increase was observed, followed by higher rates in the period from 2010 to 2015. Starting in 2016, a progressive increase in rates was observed, peaking in 2022. The predicted curve also points to a positive growth pattern in mortality rates due to intentional self-poisoning by medicines over the period. All observed values for mortality rates, except for two points in the time series, coincided with the predicted values for the nonparametric curve of mortality rates, considering a confidence level of 95%. Figure 2 suggests an absence of correlation or that the remaining noise is White, as the plotted values are close to zero.

According to Table 2, compared to 2003, in 2009, there was a slight though not significant increase in mortality rates due to intentional self-poisoning by medicines (1.15; 0.98–1.36). Compared to 2003, in 2015, there was a significant increase of 41.0% (1.41; 1.21–1.65). In 2021 and 2022, also in comparison to 2003, there was an increase of 181% (2.81; 2.44–3.23) and 264% (3.64; 3.17–4.19), respectively.

**Table 2:** Demographic features and ratios of mortality rates due to intentional self-poisoning by medicines ***, Brazil, according to the period 2003 to 2022.

Years	Deaths	Population	Mortality rate/ per one million inhabitants	Ratios	CI 95%
**2003/2009**					
2009	322	161,754,935	1.99	1.15	0.98–1.36
2003	253	146,645,947	1.73		
**2003/2015**					
2015	424	173,974,253	2.44	**1.41**	**1.21–1.65**
2003	253	146,645,947	1.73		
**2003/2021**					
2021	891	183,924,964	4.84	**2.81**	**2.44–3.23**
2003	253	146,645,947	1.73		
**2003/2022**					
2022	922	185,457,244	4.97	**3.64**	**3.17–4.19**
2003	253	146,645,947	1.73		

***X60 - Intentional overdose by exposure to nonopioid analgesics, antipyretics and antirheumatics; X61 - Intentional overdose by exposure to antiepileptic, sedative-hypnotic, antiparkinsonism and psychotropic drugs, not classified elsewhere; X63 - Intentional overdose by exposure to other drugs acting on the autonomic nervous system; X64 - Intentional overdose by exposure to other unspecified drugs, medications and biological substances.

## Discussion

There was a predominance of deaths in women, individuals aged 30–49 years, of White race/color, occurring in health facilities, that occurred with the use of medications and unspecified substances; in addition to an expressive concentration in the southern region and a positive trend in mortality rates due to intentional self-poisoning by medicines, especially from 2016 onwards.

The predominance of suicides due to intentional self-poisoning by medicines (55.5%) in women contrasts with what is observed in relation to overall suicide (by all methods), since suicide among men is consistently higher (15), both in Brazil (16) and globally (17). In line with our study, in general population from northern Tunisia, 59% of suicides by intentional self-poisoning (including all substances) occurred in women (18) and, in general Brazilian population, among the 147,698 suicides due to intentional self-poisoning by medicines, 55.7% occurred in women and 44.3% in men (16).

The factors commonly used to explain the lower mortality of women by suicide (15), as well as the preference for less violent (and in general less lethal) methods (5) and the greater demand for mental health services, may be related to the greater use of intentional self-poisoning by medicines by females. Intentional self-poisoning, for example, is considered a less violent method than hanging or the use of firearms. On the other hand, the greater demand by women for medical attention, both for mental health and for health in general, can increase access to medicines, which could favor, in vulnerable individuals, suicide due to intentional self-poisoning by medicines. Moreover, in countries with a strong influence of patriarchy such as Brazil, the role of ‘family health care manager’ falls to women (19), which can also increase their access to medicines.

Although the age group of 30–49 years represents about 34% of the Brazilian population (12), approximately half (47.2%) of suicides due to intentional self-poisoning by medicines occurred in this subgroup, suggesting premature mortality and a higher propensity of individuals aged 30–49 years toward intentional self-poisoning by medicines. Data on overall suicide (by all methods), both international (17) and Brazilian (4), indicate that the highest risk group would be the older adult.

It was also observed that more than half (53.2%) of deaths due to intentional self-poisoning by medicines occurred in White people. It should be noted that, according to the Brazilian Census of 2022, the population of self-declared Whites over 9 years of age was 43.4% and that of Blacks or Indigenous was 55.6% (20). Thus, there is evidence of a possible ethnic-racial asymmetry in the use of this method for suicide, with relatively more frequent use by Whites, compared to Blacks or Indigenous. To the extent that intentional self-poisoning by medicines is considered a less violent method of suicide, this finding aligns with findings that African Americans are 124% more likely than Caucasians to use violent methods to commit suicide (21). The Brazilian literature, excluding studies that indicate the much more frequent use of hanging by indigenous people, compared to non-indigenous people (22), does not explore ethnic-racial differences in relation to the methods used to consummate suicide, which, therefore, leaves us with an important knowledge gap.

While most suicides due to intentional self-poisoning by medicines occurred in the southeastern region (41.7%), which is the most populous region in the country and concentrated 42.3% of residents in Brazil (12), the southern region concentrated 22.8% of these deaths, but represented about 15% of the Brazilian population over 9 years of age in 2022. The southern region has the highest percentage of self-declared Whites (20) and, historically, has the highest suicide mortality rates (by all methods) in Brazil (4), both male and female (23). Such aspects may help to explain, at least in part, this higher concentration of suicides due to intentional self-poisoning by medicines in the southern region of the country. On the other hand, the northern region concentrated the lowest proportion of deaths due to intentional self-poisoning by medicines (3.7%). This value is substantially lower than its approximately 8% of the residents over 9 years of age in the country for the year 2022 (12). In this case, it is possible that greater underreporting (22, 24) and lower access to medicines (25, 26) can help to explain the disproportion observed in the northern region, compared to other regions of Brazil. Therefore, additional studies are needed in order to understand regional variations in mortality from intentional self-poisoning by medicines.

The results of our study showed that most deaths occurred in health facilities (67%). One systematic review study pointed out that, in Brazil, deaths by suicide (by all methods) occurred twice as often at home than in hospitals (27). The difference between our data and that of this review may be related to the lower lethality of intentional self-poisoning by medicines (5), as it provides a greater chance of medical care, unlike what occurs, for example, with deaths by hanging in Brazil (27).

On the other hand, a study in general population from Brazil showed that only 37.1% of deaths from suicide by intentional self-poisoning (by all substances) occurred in hospital (28). A study with data from Europe, North America, Oceania and Asia countries showed that suicide by to intentional self-poisoning (by all substances) occurred mainly at home, except in Mexico and South Korea, where the main place of death was the hospital, with 58.6 and 70.1%, respectively (29).

It is also worth highlighting that in our sample we only included suicides due to intentional self-poisoning by medicines, excluding suicides by intentional self-poisoning related with narcotics and psychodysleptics, organic solvents and pesticides, for example, which are highly lethal substances and usually make it impossible to access the hospital environment. In this regard, because we used data related to the place of death and not necessarily the place where the intentional self-poisoning occurred, it is possible that some victims may have failed in its attempt outside the home and died in hospital, for example (29).

Therefore, this finding suggests the need for constant improvement of pre-hospital and hospital care protocols in cases of intentional self-poisoning by medicines, especially in emergency department (30), given the potential to reduce mortality. Furthermore, considering serious implications of intentional self-poisoning by medicines, additional studies are necessary to better understand associated factors and improve the timely access to healthcare, especially for high-risk groups (31) but also for unintentional injury and other causes of premature mortality (32).

In our study, the main code recorded in deaths from intentional self-poisoning was X64 (54.8%), i.e., from unspecified medicines, which is similar to what was found in previous national studies on deaths from self-poisoning by medicines (regardless of intentionality) by medicines (8, 33). This type of code indicates that it was not possible to identify the substance used or that this substance does not appear in the other categories of medicines that make up the International Classification of Diseases. Therefore, our findings, on the one hand, reinforce the limitations related to the quality of filling in causes of death form on the mortality information system in Brazil, despite improvements in the last two decades (34). However, on the other hand, the limited toxicological classification of medications based on the ICD-10 (7) impairs the formulation of strategies for preventing mortality due to suicide by intentional self-poisonings.

The second most used code was X61 (40.4%), psychotropic medicines in general, one of the important pillars of control and treatment in mental health services. In this sense, it should be noted that adequate adherence to the use of pharmacological substances, especially antipsychotics, is associated with a reduction in the risk of suicides, especially in the most severe cases (35). There is also a population study that suggests that the increase in the prescription of antidepressants in young people was not associated with a decrease in mortality due to suicide (36). Despite advances in mental health care in Brazil in recent decades, important deficiencies remain, especially in outpatient care. Thus, the recurrence of long intervals between consultations may result in the prescription of large amounts of medication. On the one hand, this impairs the effective follow-up of patients and, on the other, it favors the use of psychotropic medicines for intentional self-poisoning, either by the person for whom the medication was prescribed or by third parties, especially in the absence of adequate articulation between specialized mental health services and primary health care (17).

Finally, our data suggest a clear and consistent trend of increasing mortality rates due to intentional self-poisoning by medicines in Brazil, with some inflections over time. Different factors may explain this trend. A first point to be considered would be the progressive improvement in the capacity of the mortality information system to record deaths due to intentional self-poisoning, especially in the first years of the investigated period (2003–2009), which was marked by a slight increase in mortality rates and also by an important reduction in unnecessary codes in vital statistics in Brazil (37).

Another possible explanation would be that the increase in rates of intentional self-poisoning by medicines could also be related to the trend of increasing suicide rates in general (by all methods), as some studies have shown (4, 38, 39). This understanding is corroborated by the comparison of the mortality rate due to intentional self-poisoning by medicines in 2015, in relation to 2003, which indicates an increase of 41.0% in our study, while in the comparison of suicide rates in general in Brazil, between 2000 and 2016 (4), an increase of 41.4%.

However, it is important to note that the above hypothesis seems to be insufficient to justify the clear increase in rates due to intentional self-poisoning by medicines observed from 2016 onwards since, in the period from 2015 to 2021, there is no evidence of substantial changes in the trend of mortality from suicide in general in Brazil (40). Thus, other hypotheses should be sought to understand the sudden rise in mortality rates due to intentional self-poisoning by medicines in Brazil, starting in 2016, especially in 2022, which was approximately double the rate of 2015.

It is possible that, from 2016 onwards, a set of factors may have had a more forceful impact on the specific increase in mortality rates due to intentional self-poisoning by medicines in Brazil, including the complex social, political and economic context of the period, since from 2015 and 2016 a major economic and political crisis set in (41, 42). In this biennium, there was a slowdown in the gross domestic product and the country entered an economic recession, with falls in the average worker’s income and a growth in the level of employment (43). Amid this complex scenario, in 2016, the deposition of the president of Brazil took place, giving way to governments that prioritized policies of fiscal austerity and a controversial labor reform in a context of inflation and reduction in health investments (43–45).

Figueiredo et al. (43) investigated the possible effects of the economic crisis on mortality by suicide in people over 25 years of age in Brazil and observed a slight increase in these rates for the population as a whole, from 2015 onwards, as well as a substantial increase in sub-analyses that included the victim’s geographic region of residence, sex, color or race and education level, for example, such as suggested in other Brazilian suicide trend studies (23, 46). Therefore, it seems plausible to assume a possible effect of the economic and political crisis on the increase in mortality rates due to intentional self-poisoning (47) by medicines in Brazil.

Another factor that may have contributed to the significant increase in mortality due to intentional self-poisoning by medicines, would be the increased access to medicines in the period. It is important to highlight that, despite economic crises, the drug retail sector has shown significant growth in Brazil, with record revenues (48). The sector has expanded online sales, promoting special offers, in which when buying two boxes of medicines, the customer takes a third box for free, among other initiatives to increase profits in the sector. It is important to highlight the sale, in Brazil, of anti-inflammatory medicines such as paracetamol, one of the best sellers in the country (49, 50), without any special control. This particular drug has significant toxicity and high lethality at high doses (51). In addition, it is noteworthy that, despite the economic crisis and the fall in the purchasing power of the population, there was an increase in the sale of antidepressants in Brazil during this period of economic recession (52) and these can be used for both treatment and intentional self-poisoning.

Finally, we highlight the pandemic period in Brazil, in particular the mortality rates due to intentional self-poisoning by medicines in 2021 and 2022, which were, respectively, 1.8 and 2.6 times higher than those of 2003. In Brazil, the pandemic year of 2021 was the most critical in terms of mortality (53), and was marked by deep uncertainties and suffering (54), increased self-medication (55) and a decrease in the regular supply of mental health services (56, 57), as well as evidence of increased domestic violence against women (58). This may be potentially related to the increased risk of intentional self-poisoning by medicines in this subgroup, mainly psychotropic medicines, as was the case in Croatia during the COVID-19 pandemic, for example (59). Although the COVID-19 pandemic does not seem to have resulted in a significant excess of deaths by suicide (considering all methods) in the Brazilian population as a whole (40) and yes in certain subgroups, specific analyses for suicides due to intentional self-poisoning by medicines seem necessary, mainly if we consider the atypical peak of this rate in the year 2022, which may reflect part of the indirect effects of the COVID-19 epidemic.

This study has some limitations and, therefore, our results should be interpreted with care. Our estimates may have been affected by underreporting, especially in the northern region, where the frequency of suicides due to intentional self-poisoning by medicines was substantially lower, when compared to the central-western region, which has a similar population size (24, 34). Due to the apparently limited number of suicides due to intentional self-poisoning by medicines in regions such as the north, it was not possible to explore more specific models such as by sex or age groups, for example. Another limitation was the significant proportion of deaths coded as X64, which not only limits part of the interpretation of the data, but also the adoption of policies for monitoring and restricting access to medicines related to deaths due to intentional self-poisoning (10).

As a strong point of this study, we highlight the unprecedented evaluation of data from one of the most populous countries in the world and one of the hardest hit by the Covid-19 pandemic. Our study also made it possible to identify little-explored sociodemographic characteristics of victims of intentional self-poisoning by medicines, which are substantially different from the profile of overall suicide victims (by all methods), and which may contribute to the improvement of specific preventive strategies.

## Conclusion

Finally, our work enabled not only a detailed description and evaluation of the temporal trend of suicide due to intentional self-poisoning by medicines in Brazil, but also reflections on the pharmaceuticalization of daily life and suffering, especially in conjunction with regional and global crises. Therefore, the expansion of access to medicines, either as a public health strategy to deal with health problems or as a consequence of the unbridled expansion of the pharmaceutical industry, especially in developing countries, should pay attention to possible reflections on the rise in mortality due to intentional self-poisoning by medicines.

## Data availability statement

Publicly available datasets were analyzed in this study. This data can be found at: https://datasus.saude.gov.br/transferencia-de-arquivos/. The raw data supporting the conclusions of this article will be made available by the authors, without undue reservation.

## Ethics statement

Written informed consent from the patients/participants or patients’/participants’ legal guardian/next of kin was not required to participate in this study in accordance with the national legislation and the institutional requirements.

## Author contributions

MLPS: Conceptualization, Investigation, Methodology, Validation, Writing – original draft, Project administration. JDYO: Conceptualization, Investigation, Methodology, Validation, Writing – original draft, Data curation, Formal analysis, Funding acquisition, Resources, Software, Visualization. FOJ: Conceptualization, Data curation, Investigation, Software, Validation, Visualization, Writing – original draft. BLH: Conceptualization, Formal analysis, Funding acquisition, Investigation, Methodology, Supervision, Validation, Visualization, Writing – original draft.
